# Micromanagement in Healthcare: A Narrative Review of Antecedents, Consequences, and Mitigation Strategies

**DOI:** 10.3390/healthcare14131995

**Published:** 2026-07-05

**Authors:** Maisa Hamed Al Kiyumi, Zalikha Issa Al Balushi, Rahma Al Hinai, Ahmad Al Kamli

**Affiliations:** 1 Department of Family Medicine and Public Health, Sultan Qaboos University Hospital, University Medical City, Muscat 123, Oman; 2 Department of Family Medicine and Public Health, College of Medicine and Health Sciences, Sultan Qaboos University, Muscat 123, Oman; 3 Department of Primary Health Care, Ministry of Health, Muscat 100, Oman; albelushi11@hotmail.com (Z.I.A.B.); rama.omania@hotmail.com (R.A.H.); alkamliahmed.aa@gmail.com (A.A.K.)

**Keywords:** micromanagement, healthcare, autocratic leadership, transformational leadership, professional autonomy, occupational burnout, patient safety, organizational performance, systematic review

## Abstract

**Background:** Micromanagement is an extensively prevalent yet relatively under-theorized management process in healthcare organizations. This narrative review synthesizes the literature on micromanagement and related leadership practices in healthcare, focusing on its antecedents, manifestations, consequences, and mitigation strategies. **Methods:** A structured literature search was conducted on 10 May 2024 across eight electronic databases. Eligible studies included qualitative, quantitative, mixed-methods, and applied studies published between 2003 and 2024. The main outcomes were the underlying causes and behavioral measures of micromanagement, examined directly, or closely related constructs such as excessive supervision, reduced autonomy, authoritarian leadership, toxic leadership, and controlling managerial behavior. The secondary outcomes involved organizational and patient-related effects and their respective interventions. **Results:** A total of twelve studies were selected. The identified antecedents of micromanagement were authoritarian leadership styles, autocratic and toxic leadership personality traits, overly intrusive supervisory practices, poor employee empowerment, complicated regulation, unclear definition of professional roles, and inherent structural challenges. Micromanagement behavior was seen in authoritative decision-making, transactional supervision, systematic reduction in employee autonomy, and institutionalized distrust. The consequences recorded include high levels of occupational stress, poor organizational productivity, poor quality of healthcare services, high employee turnover rates, and psychological problems. **Conclusions:** This review represents a preliminary conceptual synthesis of the literature that addresses micromanagement in healthcare. The evidence base is inconsistent, with many studies focusing on constructs that relate to micromanagement while not studying it directly. In future research, validated tools to assess micromanagement should be designed, as well as leadership interventions that benefit both workplace and patient outcomes.

## 1. Introduction

The concept of micromanagement can be understood as an approach to leadership that is marked by overly detailed supervision of employees’ actions, restricting space for making independent professional decisions, and prioritizing managerial preferences over collaboration. Micromanagement is well known as an organizational challenge but has not been extensively investigated in the scientific literature [1]. The healthcare sector offers unique conditions for micromanagement to cause harm, since professional judgment and cooperation are necessary for proper patient treatment; hence, micromanagement negatively affects the psychological state of healthcare workers [2].

The behavioral typology of micromanagement in the healthcare sector can be characterized by various dysfunctional behaviors in supervision, including obsessive scrutiny of clinical results and excessive unnecessary requests for reports on achievements, which impose personal management practices on clinical personnel, unilaterally restrain staff members’ ability to make decisions, and provoke interpersonal conflict situations with subordinates and derogatory comments toward poor performers [3,4,5,6,7,8,9]. At the organizational level, micromanagement culture is linked to deteriorating workplace trust, decreased opportunities for professional growth, and an overall decline in innovation within organizations [10]. The consequences for practitioners’ skills, mental state, and ethical principles contribute to an increased likelihood of medical errors, poor quality of treatment provided, and personnel turnover [11,12].

The existing literature describes the various consequences of micromanagement in different clinical areas. McCormack et al. (2018) found higher levels of burnout among applied psychologists due to micromanagement supervision [13]. In addition, Søvold et al. found that micromanagement is one of the most critical factors behind professional dissatisfaction and inefficiency in patient management for physicians [14]. Another body of literature demonstrates that micromanagement is linked with poor psychological outcomes, such as work disengagement and persistent psychological issues [15]. Gray et al. elaborated on the unintended organizational problems that arise when managers implement seemingly supportive programs from an overly controlling perspective [16]. Overall, these studies show that micromanagement can cause multiple organizational challenges at different levels [1,12].

There is a noticeable lack of theoretical development in the existing body of literature, thus preventing the construction of an overarching theory of healthcare micromanagement. This narrative review aims to provide a synthesis of the current healthcare literature concerning micromanagement and the related constructs of leadership and supervision. The purpose of this review is to elaborate on the concept of micromanagement, to identify its known antecedents and behavioral expressions, summarize reported outcomes, and discuss possible mitigative approaches.

## 2. Methods

### 2.1. Search Strategy and Data Sources

A comprehensive and replicable search was carried out on 10 May 2024 in eight credible electronic databases: CINAHL, PubMed/MEDLINE, Cochrane Library, Web of Science, Google Scholar, EMBASE, Scopus, and Ovid. The search employed an efficient search technique that entailed the use of both MeSH terms and text words in various combinations of Boolean operators, such as AND, OR, and NOT [17]. The full Boolean search string matrix is included in Supplementary Table S1.

Search terms combined concepts related to micromanagement and healthcare leadership, including (1) micromanagement; (2) healthcare professionals (HCP); (3) healthcare leaders; (4) clinical supervision; (5) clinical practice environment; (6) employees; (7) autonomy; (8) healthcare management; (9) leadership; (10) authoritative; (11) autocratic; and (12) transformational leadership. Boolean logic was used in different combinations to conduct topic-related searches (Supplementary Table S1).

### 2.2. Source Selection

Only sources published from January 2003 to May 2024 that addressed healthcare-related professional environments and provided evidence or conceptual insight relevant to micromanagement, over-supervision, restricted professional autonomy, authoritarian leadership, toxic leadership, or controlling managerial behaviors were considered for inclusion. Given that studies focusing on micromanagement in the context of healthcare remain limited, empirical qualitative, quantitative, cross-sectional, longitudinal, retrospective, and conceptual sources were considered, as well as applied sources. Articles not substantively related to micromanagement or related constructs, such as reviews, editorials, opinion articles, correspondence, and source articles unrelated to healthcare, were excluded. Notably, in this review, the concept of micromanagement was defined as excess managerial control, over-supervision, restriction of professional autonomy, and authoritarian oversight, and studies examining these closely related constructs in healthcare settings were considered to be eligible.

### 2.3. Primary and Secondary Endpoints

The main objectives of this literature review were to identify (i) the antecedent factors leading to the occurrence of micromanagement in healthcare organizations and (ii) the behavioral characteristics associated with micromanagement. The secondary objectives were to identify (i) the outcomes of micromanagement in organizational, clinical, and psychological settings and (ii) possible methods for addressing micromanagement.

### 2.4. Data Charting and Narrative Synthesis

Key information was extracted from each relevant source, including author, year, design, healthcare context, construct examined, relevance to micromanagement, and the main contribution to the themes of the review. Narrative thematic analysis was used to synthesize the findings. The following four organizing themes were employed: antecedents of micromanagement, behaviors exhibited, consequences, and mitigation strategies. Since the study designs were heterogeneous and conceptual, and applied sources were included, a formal risk-of-bias assessment alongside quantitative meta-analysis was not conducted. Rather, the strengths and limitations of the evidence are taken into account throughout the review narratively.

## 3. Results

### 3.1. Study Selection

In the first round of searching, 74 documents were collected, with 47 from the CINAHL, PubMed/MEDLINE, Cochrane Library, Web of Science, and Google Scholar databases and 27 from the EMBASE, Scopus, and Ovid databases. In the second round, following de-duplication, 54 documents were assessed through title and abstract review. A total of 19 records were excluded following abstract and title screening for not matching the eligibility criteria used in the study. Thirty-five articles were selected for full-text review. After the full-text assessment, studies were excluded because they failed to adequately address the construct of micromanagement or related constructs in healthcare settings, did not present relevant data for the objectives of the review, duplicated reported findings, or did not meet the methodological eligibility criteria.

All studies and their results are described in Table 1.

### 3.2. Antecedents of Micromanagement

According to Crockett et al., in their qualitative thematic study on the perception of autonomy among residents, the exclusion of trainees’ feedback from the process of making medical decisions and the tendency of attending physicians to oversee the daily routine activities of residents have been systemically reported [18]. Similarly, according to Santen et al., micromanagement was found to be an inherent characteristic of the supervision provided by resident faculty members and was considered dispositional in nature [19].

Restrictive care plans were found by Hawkins et al. in their longitudinal study on practices relating to end-of-life care among terminally ill patients [20]. Abdelaliem et al. found that the most common source of micromanagerialism within organizations was the presence of toxic leaders, which is further compounded by authoritarian management styles—a common proximate cause of micromanagement [21].

Rollins et al. highlighted the emphasis on regulatory adherence and managerial control over the well-being of clinicians in mental health institutions, which creates an organizational environment conducive to the emergence of micromanagement [22]. Cernega et al. offered one of the most comprehensive analyses of micromanagement precursors, including volatile regulatory climates, pressures towards stopping treatment, financial problems, differences in medical education standards, misinformation created in the media, and professional disagreement, all within the VUCA (Volatility, Uncertainty, Complexity, Ambiguity) context of current healthcare organizations [23].

Further antecedents that emerged within the selected articles include inconsistent availability of supervision during patient care activities [24], structural disempowerment of employees [25], organizational culture of authoritarian management [26], poor instructional supervision skills among attending physicians [27], and organizational acceptance of surveillance management [28].

### 3.3. Behavioral Manifestations of Micromanagement

Dimensions involved in the behavioral pattern of micromanagement, according to the selected studies, included excessive supervision, limited delegation of authority, constant monitoring of employee performance, overcontrol of decision-making, and reduced employee autonomy. For example, Crockett et al. and Santen et al. highlighted how residents were systematically deprived of their autonomy via authoritative supervision [18,19]. According to Hawkins et al., there was systematic adherence to protocols in advance care directives despite patients’ preferences, indicating procedural micromanagement [20]. Abdelaliem et al. described micromanagement behavior as involving threats to communication, interpersonal hostility, and the intentional organizational silencing of nurses [21]. Rollins et al. and Spector et al. described autocratic behaviors that micromanaging leaders exhibit, such as decision-making without consultation, defiance of employees’ suggestions, and restriction of professional autonomy [22,29].

Professional immaturity, lack of adaptive skills, and inadequate transversal competencies were among the behavioral traits identified by Cernega et al. as being associated with micromanagerial tendencies [23]. The use of sporadic non-constructive audits and evaluations for supervisory purposes was outlined by Grant and Wheatley (2014) as micromanagement actions [24]. Transactional leader characteristics were described by Elshout et al. as a necessary component of the structure–behavior relationship in the context of clinical care delivery organizations [26]. The purposeful suppression of resident autonomy, clinical judgment, and medical competency by their respective attending doctors was noted by Farnan et al. [27]. Finally, the behaviors of stubbornness, lack of social acceptance, power-driven motivations, disrespect to subordinates, and autocratic management practices were highlighted by Samakao and Mulenga [28].

### 3.4. Micromanagement Impact

The organizational and clinical implications of micromanagement described in the articles selected for inclusion are diverse and multifaceted. In terms of individual workers, some of the outcomes reported were reduced professional accountability and responsibility among residents [18]; de-professionalization and decreased trust among emergency medicine trainees [19]; occupational burnout, alongside enhanced work engagement in reaction to trauma, among mental health practitioners [22]; and higher psychological stress, demotivation, and counterproductive workplace behavior among frontline employees [28].

From an organizational perspective, micromanagement led to the deterioration of organizational outcomes due to the silencing of nurses [21], suppression of democratic organizational processes, and the maintenance of a work environment under stress and absenteeism, along with decreased worker satisfaction within mental healthcare facilities [26]. Moreover, it also resulted in the promotion of resistance attitudes towards supervisors by trainees [27].

At the patient level, there were significant implications for enabling patients to make independent decisions at the end of their lives [20], higher instances of pre-operative fasting among children having day surgeries due to the micromanagement of nurses [25], and an overall degradation of the interpersonal, communication, and medical aspects of patient-centered care [23,24].

### 3.5. Mitigation Strategies

Common strategies informed by evidence were identified across all included studies as viable counteractive measures against micromanagement practices within healthcare settings. According to Crockett et al., significant changes in two-way communication between the supervisor and the trainee, an increase in decision-making engagement among trainees, and institutionalized professional independence norms for trainees were suggested as effective strategies against micromanagement [18]. Similarly, the introduction of laissez-faire management strategies was proposed by Santen et al., contingent on the capability of emergency medicine residents [19].

Hawkins et al. stressed the need for shared decision-making architecture and acknowledgment of the patient’s surrogate as part of advanced care planning situations [20]. Abdelaliem et al. pointed to the need to transform the organizational culture in terms of eliminating toxic leadership and establishing psychologically healthy environments for working, along with effective communication lines [21].

Rollins et al. suggested that the application of patient-centered care models, reduction in bureaucratic regulations, and professional development of healthcare practitioners should be considered [22]. Spector et al. pointed to the importance of institutionalizing democracy in management and empowering clinicians as solutions to the micromanagement problem [29]. Cernega et al. highlighted the significance of strengthening communication between physicians and patients and using a macromanagement approach [23].

The most convincing example of this was demonstrated by Elshout et al., who found that transformational leadership can function as a structural substitute for transactional micromanagement and can enhance employee happiness, reduce absenteeism, and increase organizational commitment within mental healthcare institutions [26]. Farnan et al. advised creating theory-based supervision faculty models as an institutional approach to keeping supervisory actions in check [27]. Additionally, the use of structured socialization and positive interpersonal connections between managers and their subordinates has been overlooked as an effective solution to micromanagement issues [28].

Organizations also need to recognize the difference between necessary accountability systems and harmful surveillance. Data systems, audit processes, and protocols can contribute to safety and quality when used in a clear and constructive way, but can be a source of micromanagement if used mainly for monitoring purposes or excessive control.

## 4. Discussion

This narrative literature review offers an early synthesis of the healthcare literature concerning micromanagement and associated supervisory and leadership constructs. Notably, the review conclusions do not equate all forms of supervision, protocol compliance, or regulated clinical oversight as “micromanagement,” but view micromanagement as excessive or unnecessary oversight that denies professional autonomy beyond what is required for maintaining patient safety, patient education, and professional accountability.

The consistent conclusions drawn from 12 methodologically heterogeneous sources suggest a theoretical approach that treats micromanagement not as a subjective managerial issue but, rather, an organizational problem with profound implications for employees’ welfare, organizational efficiency, and medical service quality [1] (Mookerjee et al., 2022).

In terms of antecedents, the framework proposed in this review situates healthcare micromanagement in relation to dispositional leadership antecedents, such as autocratic or toxic management styles, and structural systemic antecedents, such as regulatory complexity, resource limitations, and inadequate training in supervising skills [18,19,20,21,22,23,24,25,26,27,28,29]. Specifically, the theoretical contribution of Cernega et al., which places micromanagement in the broader context of a VUCA (volatile) environment is notable, as this study emphasizes the impact of the instability of organizations on their managerial staff’s behavior [23]. From the perspective of such a framework, micromanagement may be more often an adaptive response to a risky environment than an intentional action, which is a crucial insight for interventions.

The commonality and structural entrenchment of autocratic leadership as an antecedent to micromanagement is deserving of special academic and practical consideration. The following discussion is an integration of findings from studies involved in this review, complemented with selected studies from the broader leadership literature, which provides context and intent for understanding review outcomes. References not used in the final synthesis sample appear as contextual evidence only, and are not a part of the formal synthesis.

Evidence from cross-cultural research shows that the phenomenon of autocratic leadership exists within healthcare institutions despite varying institutional and sociocultural settings. Autocratic leadership was noted by Blaževičienė et al. to be a dominant style used by healthcare practitioners in a regional hospital in Lithuania amid the country’s healthcare system transformation period [30].

Autocratic tendencies were also found in mid-level nurse leaders in Qatar, regardless of ongoing institutional interventions aiming at adopting transformational leadership models, which indicates that leadership culture transformation is characterized by high structural inertia, as evidenced by Al-Thawabiya et al. [31]. This suggests that leadership style, as an adjustable factor contributing to healthcare micromanagement, has relevance across contexts.

Micromanagement is more likely to occur in a hierarchical, top-down organizational structure where decision-making is highly centralized, authority is concentrated at senior management levels, and there is limited delegation of authority. While hierarchical structures are crucial in healthcare to protect patient safety, hold people accountable, and ensure adherence to healthcare regulations, excessive managerial control can also limit professional autonomy, undermine psychological safety, and hinder innovation [32,33]. In contrast, leadership styles that foster shared decision-making, empowerment, and distributed leadership may be effective at reducing micromanagement without compromising the quality of clinical governance and patient care provision [33].

As noted in the literature, the effects associated with autocratic leadership are low levels of job satisfaction, rapid employee burnout, higher rates of turnover, decreased clinical innovation, and poor team cohesion [34,35,36]. This list of outcomes associated with micromanagerial behavior is entirely congruent with those observed with respect to autocratic leadership. It should be noted that there are also specific outcomes associated with micromanagerial behavior that have clinical relevance; in particular, research has found a positive correlation between autocratic and micromanagerial leadership and an increase in medical errors and patient safety issues resulting from poor team communication and staff psychological safety [21].

The behavioral indicators of micromanagement observed in the current analysis—including the behavior of an authoritarian supervisor, transactional leadership style, subordinates’ disempowerment, and organizational mistrust—fit well into the conceptualization of self-determination theory (SDT), developed by Ryan and Deci. Specifically, this theoretical approach is based on the notion that, if the basic psychological needs of autonomy, competence, and relatedness are not met, the individual experiences serious negative motivational, emotional, and behavioral outcomes [37,38]. In other words, SDT can be applied as a meta-theoretical perspective to explain the dynamics and consequences of micromanagement.

The mitigation measures identified in this literature review have two evidence-based concepts in common: the concept of professional autonomy as an organizational structure, and the adoption of transformational leadership as a preferred form of management style. The concept of transformational leadership is defined by the ability of managers to consider the development needs of their followers, inspire them, stimulate their creativity, and provide ideal leadership. This management style is well suited to replacing the transactional and autocratic approach that is often found in micromanagement environments [37].

The results obtained from this review have several implications for healthcare organizations, medical and nursing education, and health policies. For healthcare institutions, an assessment should be made regarding supervisory practices in terms of their compatibility with professional autonomy requirements and the provision of training that is based on empirical evidence for developing transformational and autonomy-supportive leadership qualities. In the context of medical and nursing education, the inclusion of the issue of micromanagement in the training program of future supervisors will provide students with sufficient knowledge and skills to develop their subordinates’ professional competence without limiting their autonomy.

From a health policy perspective, implementing supervisory quality measures as part of the hospital and departmental quality and safety process, supported by leadership governance, performance management, and quality improvement systems, is recommended as a means of promoting supportive leadership practices and safeguarding professional autonomy.

## 5. Limitations

There are a number of limitations in this review. First, the literature that directly focuses on micromanagement in healthcare is limited, and some of the literature sources address related constructs instead of micromanagement as a primary outcome. Second, the accompanying literature is methodologically heterogenous, including qualitative, quantitative, mixed-methods, conceptual, and applied studies. Therefore, the findings of this review must be considered as a preliminary conceptual synthesis and not as proof of causation. Third, differences in study design, populations, constructs, and outcomes preclude the possibility of meta-analysis. Fourth, since there was a mix of conceptual and applied studies in addition to empirical research in the evidence base, this narrative review did not utilize a design-specific formal risk-of-bias assessment of all sources. Finally, the terminology around micromanagement remains inconsistent, which may have influenced how relevant studies were identified and interpreted.

## 6. Conclusions

This narrative review offers an early synthesis of the healthcare literature pertinent to micromanagement and associated managerial practices. The findings indicate that excessive managerial control and restrictions on professional autonomy can have negative effects on health professionals, organizational performance, and patient care, but the current evidence is limited and inconsistent. Autocratic and dysfunctional leadership behaviors appear to be consistently identified risk factors, while transformational leadership and improved interprofessional communication are the most consistent mitigating factors.

The current findings offer a preliminary framework for healthcare providers, educators, and policymakers who are interested in understanding and combating micromanagement in the healthcare context. In future research, particular emphasis should be placed on: (i) the creation and validation of healthcare-oriented tools to measure micromanagement; (ii) designing and evaluating multilevel, theory-based programs to tackle micromanagerial supervision cultures; (iii) longitudinal and prospective studies to assess the organizational and patient implications of adopting a transformational leadership style as a preventive tool against micromanagement; and (iv) cross-country comparisons aimed at identifying the interaction between different cultural, structural, and personal determinants of micromanagement.

## Figures and Tables

**Table 1:** Study characteristics and findings.

Authors	Study Type	Methods	Micromanagement				Inferences
			Antecedents	Behavior	Impact	Solutions	
Crockett et al. 2019 [18]	A qualitative survey study	Qualitative thematic analysis of the autonomy/micromanagement perspectives of the resident physicians	The medical decision-making process was devoid of input from resident physicians Excessive involvement of attending physicians in the day-to-day workings of the resident physicians	Undermining the autonomy of residents by the attending physicians	Reduction in the sense of responsibility among the resident physicians	Clear communication with resident physicians to enhance their autonomy Encouragement to resident physicians to provide their input Promotion of independent activities among resident physicians No interference of attending physicians in the thought processes of resident physicians	The micromanagement of resident physicians by the attending physicians restricts their autonomy and patient care capacity Resident physicians should be encouraged to manage the patient care processes independently and reject any micromanagement attempts from the attending physicians
Santen et al. 2019 [19]	A qualitative study	Transcription and grounded theory-guided coding of audiotaped discussions of faculty members and residents	Micromanagement temperament among the resident faculty members	An authoritative supervisory pattern	Disempowerment of residents	Implementation of laissez-faire policy for work management in the emergency department	Micromanagement proved to be a personality trait and a faculty factor impacting the autonomy and entrustment among emergency medicine residents
Hawkins et al. 2005 [20]	A longitudinal study	Questionnaires and interviews were organized for 337 outpatients (age ≥65 years) and their surrogate decision-makers Psychological assumptions regarding the Advance Directives, Values Assessment and Communication Enhancement (ADVANCE) were further evaluated by the authors	Implementation of advanced care planning-related standard approaches based on the micromanagement of patients’ preferences by themselves	Stringent implementation of the advance care directives following the standard guidelines	Challenges in making end-of-life decisions for terminally ill patients	Expanding the scope of decision-making leeway and implementation of patient perspectives regarding life-sustaining treatments could help minimize the present micromanagement in the end-of-life care processes	Participants rejected the use of micromanagement approaches concerning their end-of-life decision-making Patients did not appreciate the documentation of their end-of-life treatment preferences and rather encouraged the autonomy of surrogates to implement their treatment-related decisions
Abdelaliem et al. 2023 [21]	A quantitative, descriptive, correlational, cross-sectional study	The purposive sampling approach was used to deploy experienced nurses (*n* = 750) to participate in survey questionnaires The study endpoints included employee silence, organizational performance, and toxic leadership Employee silence was investigated via structured equation modeling	Toxic leadership	Micromanagement behaviors based on threatening attitudes and rudeness of the leaders	Reduced organizational performance, mediated by the silence of nurses	Establishment of a nurse-friendly work environment to improve organizational culture by minimizing toxic leadership and strengthening open communication	Toxic leadership significantly impacted organizational performance variance (87%) and nurses’ silence variance (65%) Organizational performance variance (73%) was mediated by the silence of nurses
Rollins et al. 2021 [22]	A qualitative randomized study, including forty mental health clinicians	Open-ended questions were administered among all participants to evaluate the facilitators and barriers to their work engagement and burnout	Prioritizing micromanagement over the employee’s well-being	Leaders aimed to reduce the autonomy of employees	Elevated work engagement and burnout	- Provision of person-centered care - Reduction in bureaucratic approaches - Improvement in self-care and professional development patterns among employees	Organizational-level improvement and reduction in micromanagement approaches are the keys to enhancing employee well-being and work engagement
Cernega et al. 2024 [23]	Conceptual research	This study conceptually evaluated management approaches to minimize ambiguity, complexity, uncertainty, and volatility in healthcare	- Treatment abandonment risk - Healthcare costs - Legal regulations - Healthcare provider’s ability - Absence of security - Limited medical service access - Contrasting opinions of healthcare leaders - Low medical education quality - Media influence/fake news - Contradictory legislation interpretations	Low professional adaptability and transversal competencies	Reductions in vision, understanding, clarity, and agility among healthcare employees and patients	Improved healthcare management with enhanced doctor–patient association Replacement of micromanagement approaches with micromanagement strategies	Enhancement of functional mechanisms in the healthcare setting is key to improving the doctor’s ability to evaluate/anticipate patient requirements and adapt to challenging patient management situations
Grant and Wheatley. 2014 [24]	Applied research	Op HERRICK 18 (IT-based system) was developed to integrate, manage, and display prehospital healthcare interventions	No regular access to prehospital healthcare activities	Intermittent and threatening audits and evaluation of healthcare interventions	Elevated control of supervisors on healthcare-related activities	Increased engagement of healthcare professionals in prehospital healthcare activities	Real-time assessment of key data, remote delivery of assurance, and better engagement of healthcare professionals are key to fostering and accommodating prehospital healthcare interventions in the clinical practice environment
Ong and Walker. 2022 [25]	A retrospective study	Kotter’s eight-step change model was implemented to minimize excessive fasting in pediatric patients eligible for day surgery	Low empowerment of healthcare staff	Micromanagement of health support strategies	Elevated over-fasting prevalence among pediatric patients	Implementation of healthcare/patient support interventions, while avoiding micromanagement	Reduction in over-fasting prevalence among pediatric patients, after reducing micromanagement of healthcare approaches
Elshout et al. 2013 [26]	A mixed-method (qualitative and quantitative) study	Semi-structured interviews were used to evaluate employee satisfaction, absenteeism, and leadership style in a mental healthcare center, with stressful work culture	Excessive control of employees by their managers, via micromanagement strategies	Transactional leadership style of managers	Elevated sickness rates and reduced satisfaction among employees	Establishment of transformational leadership approaches to improve employee satisfaction, work engagement, and overall performance	Transformational leadership, rather than micromanagement via transactional leadership, is the key to reducing absenteeism, improving sickness protocol handling, and enhancing employee satisfaction rates in the mental healthcare facility
Farnan et al. 2009 [27]	A mixed-method quantitative and qualitative study	An interview-based survey was disseminated among the internal medicine residents to inquire about their intent to supervise during various clinical situations	Extreme and ineffective supervision approaches by attending physicians	The tendency of attending physicians to reduce autonomy in resident physicians while minimizing their trust and medical competence and increasing their apathy	Increased resistance in resident physicians against the supervision tactics and micromanagement of the attending physicians	Development of a theoretical framework and a comprehensive faculty aiming to improve supervision approaches while minimizing the extent of micromanagement in the clinical practice environment	Resident physicians do not require thorough mentorship from the attending doctors; they also intend to work and act independently in patient management situations requiring direct guidance from the attending physicians
Samakao and Mulenga. 2023 [28]	A qualitative study	Systematic observations, focused group discussions, and semi-structured questionnaires were undertaken to record the micromanagement-related perceptions/interpretations of 30 participants	Excessive control and observation of employees by supervisors	Indifference behaviors and stubbornness among leaders Autocratic conduct, disrespectfulness, and power hunger among supervisors	Counterproductivity, high stress levels, and demotivation among employees	Improved socialization and positive interactions between the managers and the employees	Micromanagement adversely impacts productivity, establishes an environment of insecurity and mistrust, and counters creativity and innovation among employees
Spector et al. 2024 [29]	A mixed-method study of 273 reports concerning 44 managers	Two-week surveys aimed to gather data for analyzing the association between job satisfaction/trust of employees in their leaders and leadership behaviors	Authoritarian type of leadership	Autocratic and authoritarian behaviors, challenging the autonomy of employees	Low empowerment and the absence of a democratic working style among employees	Empowerment of employees and promotion of democratic working approaches among leaders	Professionalism, fairness, and transparency correlated with job satisfaction among employees Autonomy enhancement among employees warranted a reduction in micromanagement and autocratic/authoritarian practices

## Data Availability

All data supporting the conclusions of this review are derived from previously published studies, which are cited in the reference list. No new primary data were generated.

## Supplementary Materials

The following supporting information can be downloaded at: https://www.mdpi.com/article/10.3390/healthcare14131995/s1, Table S1: Boolean search string matrix used across the eight databases.
