# Supplementary material for: Micromanagement in Healthcare: A Narrative Review of Antecedents, Consequences, and Mitigation Strategies
Source: Healthcare (Basel). 2026 Jul 5;14(13):1995. doi: 10.3390/healthcare14131995 (PMC13361376; doi:10.3390/healthcare14131995)
Supplement: Supplementary file 1 [file healthcare-14-01995-s001.zip › healthcare-4382866-supplementary.pdf]

**Supplementary Table S1 (Search strategy)**

| | |
|---|---|
| Search string-1 | "Micromanagement” AND “HCPs” |
| Search string-2 | "Micromanagement" AND “Healthcare" professionals" OR “Healthcare leaders” |
| Search string-3 | “Micromanagement" AND “Clinical supervision" |
| Search string-4 | "Micromanagement" AND “Clinical practice environment" |

| | |
|---|---|
| Search string-5 | "Micromanagement" AND "Employees" OR “Leaders" |
| Search string-6 | "Micromanagement" AND “Autonomy"OR “Healthcare management" |
| Search string-7 | "Micromanagement" AND "Leadership" OR "Authoritative” OR "Autocratic" NOT "Transformational" |
| Search string-8 | "Micromanagement” AND "HCPs” OR “Healthcare professionals" OR “Healthcare leaders" AND "Clinical supervision" AND "Clinical practice environment" AND "Employees" OR “Leaders” AND "Autonomy” OR “Healthcare management “AND“ Leadership" OR “Authoritative" OR “Autocratic" NOT “Transformational" |
